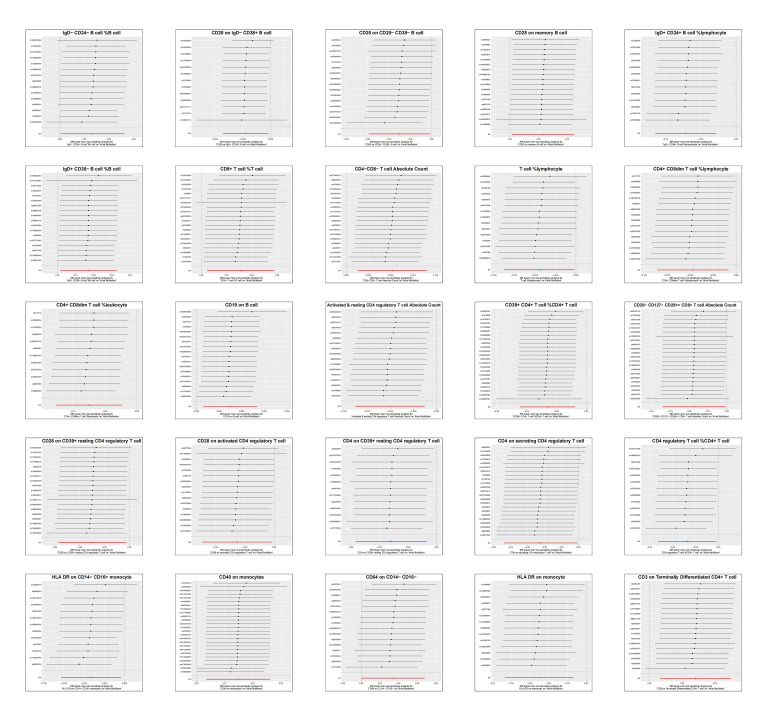# Causal relationship between immune cells and atrial fibrillation: A Mendelian randomization study

**DOI:** 10.1097/MD.0000000000038079

**Published:** 2024-05-10

**Authors:** Haoxuan Chu, Xia Guo, Hanchi Xu, Shipeng Wang, Jiahuan He, Yushi Wang

**Affiliations:** a Department of Cardiovascular Medicine, The First Hospital of Jilin University, Changchun, China.

**Keywords:** atrial fibrillation, causal associations, epidemiology, genetics immune cell, Mendelian randomization

## Abstract

Atrial fibrillation (AF) is a prevalent cardiac arrhythmia, with recent research indicating a correlation between immune system characteristics and the development of AF. However, it remains uncertain whether the immunological response is the primary underlying component or a secondary consequence of AF. Initially, we investigated the effect of immune cells on AF by performing forward Mendelian randomization (MR) analyses with immune cells as the exposure variable and their associated genetic variants as instrumental variables. Subsequently, we performed reverse MR analyses with AF as the exposure variable and immune cells as the outcome variable to exclude the interference of reverse causality, to distinguish between primary and secondary effects, and to further elucidate the causal relationship between the immune system and AF. We discovered that membrane proteins on specific immune cells, such as CD25 on memory B cells—which functions as a part of the interleukin-2 receptor—may be risk factors for AF development, with odds ratios of 1.0233 (95% confidence interval: 1.0012–1.0458, *P* = .0383). In addition, certain immune cell counts, such as the CD4 regulatory T cell Absolute Count, play a protective factor in the development of AF (odds ratio: 0.9513, 95% confidence interval: 0.9165–0.9874; *P* = .0086). More detailed results are elaborated in the main text. Our MR study has yielded evidence that substantiates a genetically inferred causal association between the immune system and AF. Identifying the risk factors associated with AF is vital to facilitate the development of innovative pharmaceutical treatments.

## 1. Introduction

The prevalence of atrial fibrillation (AF) in adults is statistically between 2% and 4%, and the incidence of AF is still on the rise.^[1,2]^ Although certain risk factors for AF such as age, male sex, and family history cannot be altered, it is imperative to conduct additional research to investigate the underlying causes and pathogenesis of AF.^[3–6]^

Inflammation is an immune response of cells or tissues. Inflammation can induce oxidative stress, fibrosis, and thrombosis, resulting in changes in cardiac electrical, structural, and autonomic neurons, thereby promoting the formation of AF.^[7–11]^ In comparison to patients with normal sinus rhythm, patients with AF exhibit elevated levels of inflammatory markers in their blood, including CRP, HSP β1 (also known as HSP27), IL-6, IL-8, and TNF.^[9,11–15]^

Previous clinical studies have established a correlation between immunity and AF.^[16–18]^ However, the capacity of observational studies to address potential confounding factors and mitigate reverse causality is limited. Mendelian randomization (MR) can be considered as an alternative to randomized controlled trials that use genetic variants to show causality between exposure and outcomes.^[19]^ It utilizes the random assignment of genetic variation as an instrumental variable (IV), effectively addressing issues related to reverse causality and confounding factors that could otherwise obscure the results.^[20]^ This study aimed to explore the interaction between the immune system and AF through the analysis of comprehensive data from genome-wide association studies (GWAS).^[21]^

## 2. Materials and methods

### 2.1. Research methodology

**Figure 1.**
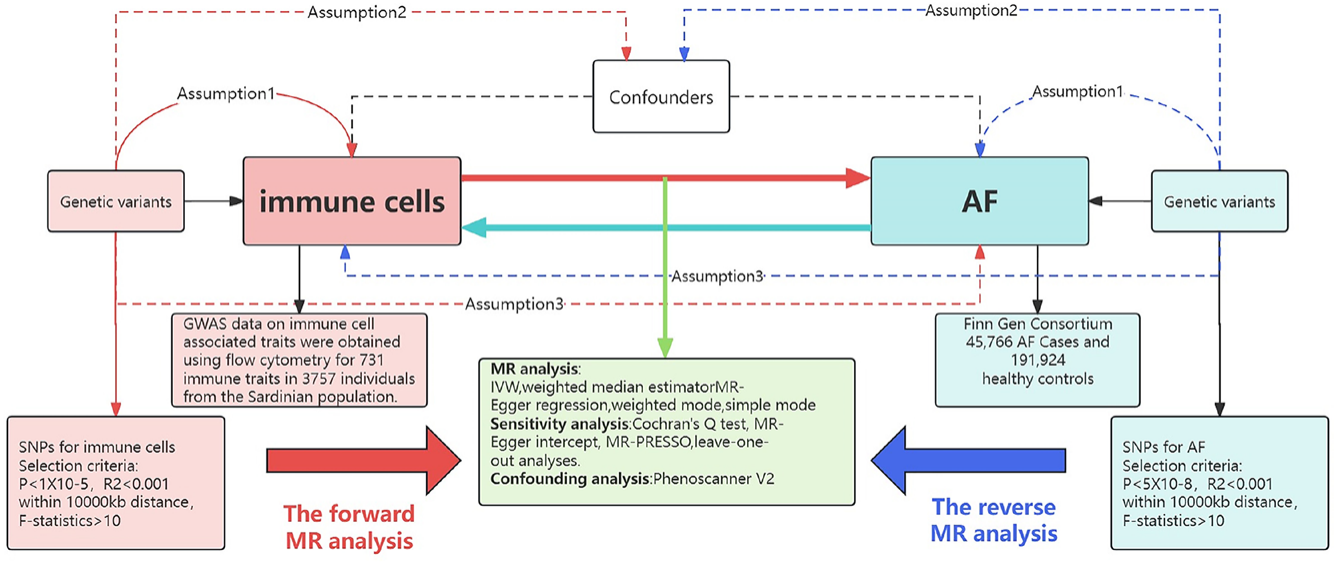
Design of this study. AF = atrial fibrillation, GWAS = genome-wide association study, MR = Mendelian randomization, SNP = single nucleotide.

We conducted a 2-sample MR analysis to comprehensively investigate the causal relationship between 731 immune cell features categorized into 4 groups and AF. The utilization of genetic variation in MR to depict risk factors necessitates the employment of validated IVs that adhere to 3 crucial assumptions for dependable causal inference: the direct correlation of genetic variation with exposure; the absence of correlation between genetic variation and potential confounders between exposure and outcome; and the assurance that genetic variation does not impact the outcome through pathways unrelated to exposure.^[22]^ The experimental design of this study is shown in Figure 1.

### 2.2. Source of data

#### 2.2.1. GWAS data sources for AF

Summary-level statistics for AF were extracted from the latest release of the Finn Gen GWAS results (R9 release), with the corresponding phenotype coding tag finngen_R9_I9_AF. This dataset consisted of 45,766 patients who had received a diagnosis of AF based on International Classification of Diseases codes (ICD-10: I48). To minimize any potential overlap in the samples used for the GWAS of AF and to minimize the risk of type 1 errors, an additional 191,924 control individuals were included. The Finnish Genetics Study is an ongoing international scientific study that aims to collect and analyze genetic and health information from 500,000 individuals in Finland. As of now, a total of 224,737 participants have been successfully enrolled.^[23]^ For more detailed information on FinnGen Consortium, please refer to the FinnGen documentation available at https://finngen.gitbook.io/documentation/v/r9/.

#### 2.2.2. GWAS data sources for immune cells

This study derived statistics from a GWAS involving 3757 Sardinian participants, aiming to explore the impact of natural genetic variations on immune-related traits and characteristics of blood immune cells. It analyzed 731 immune traits, encompassing 118 absolute cell counts, 389 mean fluorescence intensities of surface antigens (a quantitative test for surface antigens), 32 morphological parameters, and 192 relative counts. Out of 459 cellular traits across 69 loci, the research identified 122 significant, independent association signals, of which 52 were novel. The comprehensive analysis of approximately 22 million variants across 731 immune cell traits elucidated multiple molecules and mechanisms of cellular regulation, providing new ideas for identifying potential drug targets in specific immune cell pathways.^[21]^

### 2.3. Choice of IVs

In our MR analyses, we set out to evaluate 731 immunophenotypes. SNPs closely associated with immune cell characteristics were pinpointed and will subsequently be used as IVs. To guarantee the validity and precision of the inferred causal relationship between immune cell characteristics and AF, we applied stringent criteria for selecting our IVs: we prioritized SNPs with a phenotypic correlation of *P* < 1 × 10^−5^. This threshold was chosen to account for the fact that immune cell motifs identified by GWAS rarely reach conventional genome-wide significance levels (*P* < 5 × 10^−8^), which allows us to include a wider range of genetic variants that may be associated with immune phenotypes. Although the criteria for immune cells as IVs are relatively lenient (*P* < 1 × 10^−5^), they have been utilized in multiple MR studies.^[24–27]^ Using the European samples of the 1000 Genomes Project as our reference panel, we calculated linkage disequilibrium between the identified SNPs. SNPs with *R*^2^ < 0.001 within an aggregation window size of 10,000 kb and exhibiting minimal *P* values were retained. Systematic discarding of SNPs with a minor allele frequency of 0.01 or lower. In instances where palindromic SNPs were identified, we inferred alleles on the forward strand based on allele frequency data. The robustness of each SNP was evaluated using the *F* statistic, defined as *F* = *R*^2^ × (*N* − *K* − 1) ÷ [*K* × (1 − *R*^2^)]. Here *R*² denotes the fraction of exposure variance due to genetic variation, *N* denotes the size of the sample, and *K* denotes the count of IVs. *R*^2^ = 2 × (1 − EAF) × EAF × (β/SD)^2^, where minor allele frequency is the minor allele frequency, β is the allele effect value, and SD is the standard deviation. An *F*-statistic of 10 or higher indicated that the SNP possessed sufficient validity for the trait under study. Conversely, SNPs with an *F*-statistic < 10 were deemed weak IVs and consequently excluded from our analysis. Furthermore, to further ensure the quality of our selected SNPs, we utilized the Phenoscanner database to identify and exclude potentially confounding SNPs that might be causally related to AF.

### 2.4. Statistical analysis

#### 2.4.1. MR analyses

In this study, we used multiple MR methods to elucidate the causal relationship between immune cells and AF, including inverse variance weighting (IVW), weighted median estimator, MR-Egger regression, weighted mode, and simple mode. In the context of multiple IVs, predominant MR studies have employed IVW. This method optimizes the weights assigned by inverting the variance, thereby allocating greater weight to studies characterized by lower variances. Consequently, IVW enhances the estimation of the true effect size and augments statistical efficiency.^[28]^ Even under weaker assumptions, the MR-Egger regression robustly estimates causal effects, thus increasing statistical flexibility.^[29]^ The weighted median estimator assigns weights to genetic variants based on their strength and validity as IVs, thereby diminishing the influence of null instruments. This approach ensures reliable causal estimation of effects, even when up to 50% of the analyzed data originates from genetic variants with null IVs.^[30]^ The additional methodology provides depth and breadth to ensure a comprehensive assessment of our hypotheses.

#### 2.4.2. sensitivity analysis

Given the potential for bias introduced by IVW, we conducted sensitivity analyses encompassing heterogeneity tests, directional pleiotropy, and additional sensitivity evaluations to further ascertain the reliability and stability of the MR results. Within the context of 2-sample MR analysis, we evaluated the heterogeneity of IVs using Cochran’s *Q* statistic. Significant heterogeneity is suggested when the *Q* statistic’s value surpasses the number of IVs minus one, or the *P* value falls below .05. Addressing such heterogeneity is crucial for ensuring the reliability of MR estimates and mitigating potential bias.^[31]^ We employed the MR-PRESSO technique to identify horizontal pleiotropy, which represents a possible bias in MR studies. This approach compares the observed distances of SNPs from the regression line to those anticipated under the null hypothesis of no horizontal pleiotropy. The intercept test for MR-Egger regression was also used to detect genetic pleiotropy. If the intercept is close to 0 (*P* > .05), it is less affected by pleiotropy.^[32]^ We performed leave-one-out analyses by removing each IV to discern whether causality was disproportionately affected by specific SNPs.^[33]^

#### 2.4.3. Reverse MR analysis

MR leverages the random arrangement of alleles of a genetic variant at conception to simulate the random assignment in a randomized controlled trial, using the genetic variant as an IV to establish causality between exposure and outcome. In traditional MR studies, the typical direction of analysis moves from a specific exposure to a particular outcome, with the aim of assessing whether the exposure causally influences the outcome. However, in intricate biological systems, the relationship between exposure and outcome can be bidirectional, particularly in the presence of feedback loops. This means that not only does the exposure impact the outcome, but the outcome can also reciprocally influence the level of exposure. Bidirectional MR enables the differentiation between primary and secondary effects, offering a more comprehensive approach to circumvent the confounding effects of reverse causality. The data source for the reverse MR mirrored that of the forward MR. In this analysis, AF was treated as the exposure, with SNPs closely linked to AF (*P* < 10^−8^) serving as the exposure variables. Similar to the forward analysis, a selection procedure was implemented, which included eliminating chain imbalances and IVs with an *F*-statistic less than 10. Significant genera identified in the forward MR will be utilized as outcomes in subsequent 2-sample MR analyses to investigate the causal relationship between AF and immune cells.

## 3. Result

**Figure 2.**
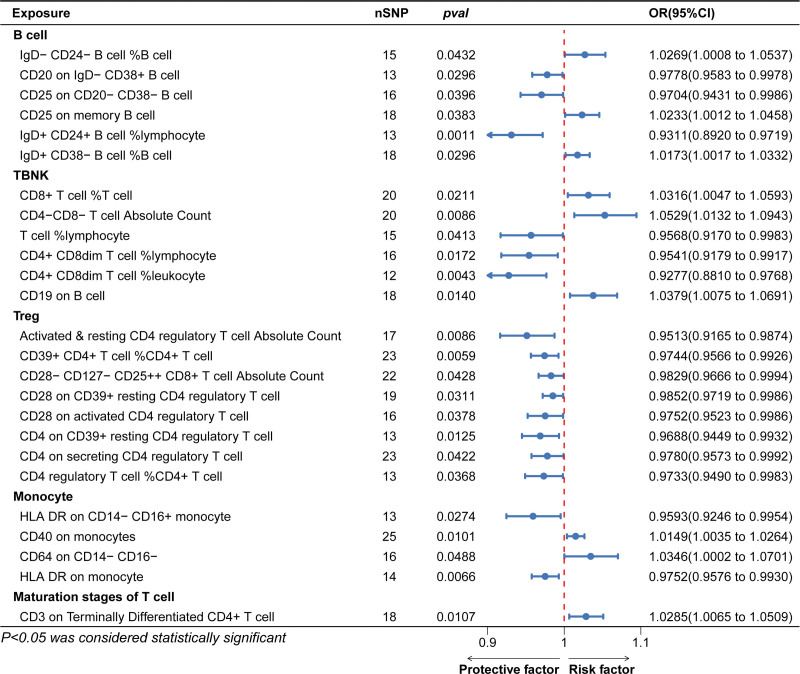
Forest plot of IVW results from a forward MR. IVW = inverse variance weighting, MR = Mendelian randomization.

**Figure 3.**
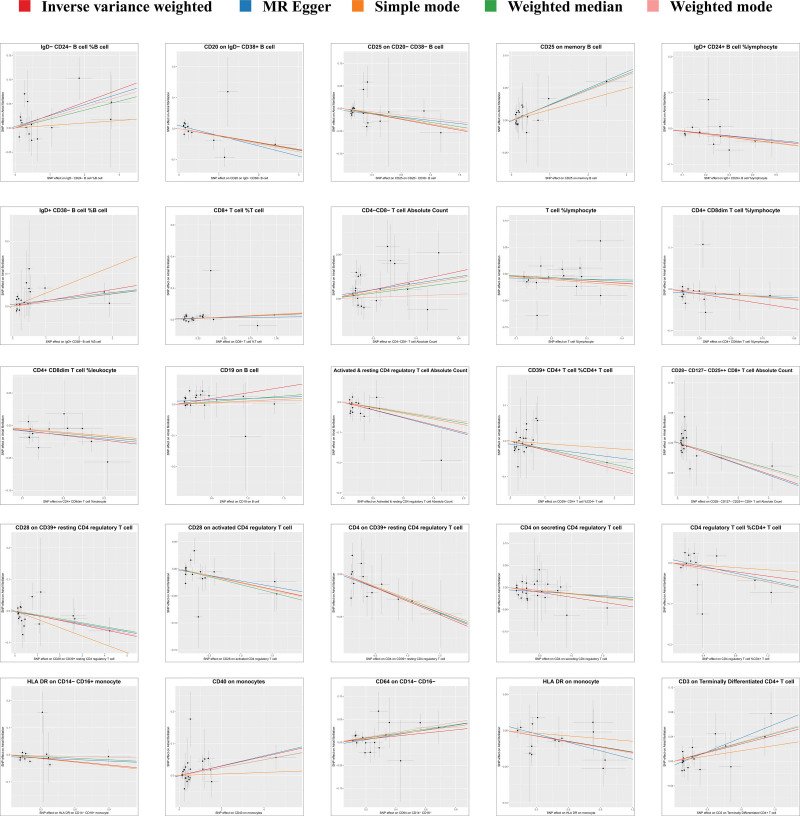
Scatterplots for forward MR. MR = Mendelian randomization.

**Figure 4.**
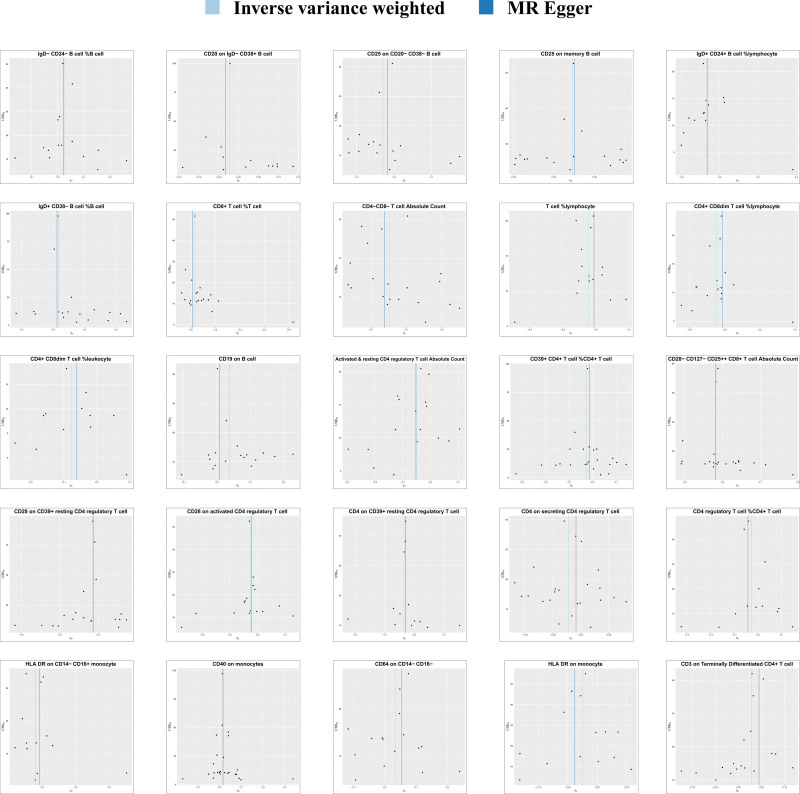
Forest plots of IVW results from inverse MR. IVW = inverse variance weighting, MR = Mendelian randomization.

A total of 14,843 SNPs were used as IVs for 731 different immunophenotypes according to the selection criteria for IVs (Details of these selected IVs can be found in Table S1, Supplemental Digital Content, http://links.lww.com/MD/M472). We found that the absolute and relative counts of certain immune cells and the expression of certain antigens on immune cell membranes were strongly associated with the development of AF. According to the IVW method, the expression level of CD19 on B cells, absolute CD4^−^CD8^−^ T cell counts, and relative CD8^+^ T cell count levels were positively correlated with AF, with odds ratio (OR), 95% confidence interval (95% CI), and *P* value of (OR: 1.0379, 95% CI: 1.0075–1.0697; *P* = .0140), (OR: 1.0529, 95% CI: 1.0132–1.0943; *P* = .0086), (OR: 1.0316, 95% CI: 1.0047–1.0593; *P* = .0211). However, the absolute CD4-regulatory T-cell count (OR: 0.9513, 95% CI: 0.9165–0.9874; *P* = .0086), the percentage of T-cells in lymphocytes (OR: 0.9568, 95% CI: 0.9170–0.9983; *P* = .0413), and the expression level of HLA DR on monocytes were significantly lower than those on lymphocytes. HLA DR expression levels (OR: 0.9752, 95% CI: 0.9576–0.9930; *P* = .0066) were protective against AF. The main MRI results are shown in Figure 2, and the results of all Mendelian randomization methods can be seen in Table S2, Supplemental Digital Content, http://links.lww.com/MD/M473. MR-Egger regression weighted median estimator, simple model and weighted model also support our arguments (Fig. 3). The results of the leave one out analyses suggest that our conclusions are not affected by a single SNP (Figure S1, Supplemental Digital Content, http://links.lww.com/MD/M471). During the MR-PRESSO global test, all significant results consistently yielded *P* values higher than .05, suggesting the absence of significant horizontal pleiotropy. The MR Egger regression was consistent with the results of the MR-PRESSO global test, providing additional confirmation of the study findings. Cochran’s *Q*-test shows no heterogeneity of effects between various SNPs in immune cells. The Steiger’s test revealed a statistically significant *P* value below .05, suggesting a robust correlation between the IV and the immune phenotype. This finding reinforces the accurate directionality of the causative effect and bolsters the reliability of employing MR to elucidate the causal association between immune cells and AF. Table 1 presents the outcomes of the heterogeneity test, the horizontal pleiotropy, and the Steiger test. The same MR method was used to perform a reverse study of the causal relationship between AF and the immune system, the results of which showed that no reverse causality was observed, which avoids the interference of reverse causality. The results of the reverse MR are shown in Figure 4.

**Table 1:** Summary of sensitivity analysis results.

Exposure	Heterogeneity test		Pleio test		Steiger test	MRPRESSO_GLOBAL (*P* value)
	Cochran’s_*Q*	Cochran’s_*Q (P* value)	MRegger_intercept	MRegger_intercept (*P* value)		
IgD-CD24^−^ B cell %B cell	18.3846	.1898	0.0029	.6972	1.72E-73	.264
CD20 on IgD-CD38^+^ B cell	17.3906	.1355	0.0099	.0928	9.83E-60	.315
CD25 on CD20^−^CD38^−^ B cell	12.4722	.6430	−0.0044	.4282	1.26E-77	.622
CD25 on memory B cell	5.7909	.9945	−0.0011	.8171	6.81E-150	.998
IgD^+^CD24^+^ B cell %lymphocyte	7.6964	.8084	−0.0017	.8533	8.00E-78	.825
IgD^+^CD38^−^ B cell %B cell	17.2751	.4359	0.0061	.2155	1.22E-88	.573
CD8^+^ T cell %T cell	21.7496	.2969	0.0074	.1811	7.56E-97	.347
CD4^−^CD8^−^ T cell Absolute Count	23.9773	.1970	0.0028	.7173	1.05E-109	.229
T cell %lymphocyte	18.2864	.1940	−0.0067	.6093	1.11E-89	.23
CD4^+^CD8dim T cell %lymphocyte	14.0323	.5231	−0.0072	.3075	2.95E-91	.596
CD4^+^CD8dim T cell %leukocyte	7.7522	.7353	−0.0021	.8720	2.98E-71	.766
CD19 on B cell	16.6093	.4811	0.0104	.0875	9.17E-87	.431
Activated and resting CD4 regulatory T cell absolute count	16.2975	.4324	0.0005	.9468	6.94E-90	.46
CD39^+^CD4^+^ T cell %CD4^+^ T cell	31.2971	.0902	−0.0087	.0930	2.34E-275	.16
CD28^−^CD127^−^CD25^++^CD8^+^ T cell absolute count	14.7388	.8358	0.0005	.8960	8.32E-110	.84
CD28 on CD39^+^ resting CD4 regulatory T cell	13.6625	.7508	−0.0027	.5508	6.94E-111	.824
CD28 on activated CD4 regulatory T cell	14.0492	.5218	−0.0019	.6941	7.03E-103	.591
CD4 on CD39^+^ resting CD4 regulatory T cell	10.3700	.5835	−0.0016	.8125	1.92E-65	.722
CD4 on secreting CD4 regulatory T cell	16.2628	.8026	−0.0055	.3480	3.08E-124	.822
CD4 regulatory T cell %CD4^+^ T cell	13.0146	.3680	0.0086	.2985	2.32E-70	.329
HLA DR on CD14^−^CD16^+^ monocyte	20.4055	.0598	−0.0067	.4711	3.39E-142	.064
CD40 on monocytes	16.2823	.8776	−0.0009	.8485	5.49E-230	.897
CD64 on CD14^−^CD16^−^	15.1560	.4402	−0.0048	.5544	2.18E-90	.439
HLA DR on monocyte	10.9357	.6162	0.0070	.3621	5.29E-238	.639
CD3 on terminally differentiated CD4^+^ T cell	12.2590	.7842	−0.0066	.1794	4.97E-110	.821

## 4. Discussion

In this research, we present evidence supporting the causal association between the immune system and AF through MR analysis from a genetic viewpoint. Recent findings indicate that inflammation actively contributes to the onset and progression of AF, rather than only observing from the sidelines.^[11,34]^ Immune remodeling is a constant occurrence that takes place during the entire phase of AF development and continuance. It causes changes in the electrical, structural, and neurological properties of the heart muscle, and triggers disease-related changes associated with AF, such as fibrosis.^[35]^ Furthermore, AF can itself induce inflammation during atrial remodeling, thereby perpetuating the arrhythmia in a phenomenon often referred to as “AF begets AF.”^[36]^ In a case-control study of 305 patients with AF and 150 control samples, IL-6, IL-8, IL-10, TNF-α, monocyte chemotactic protein -1 were independently associated with AF (all *P* values < .05).^[37]^

In a single-center prospective observational study, left atrial tissue was obtained from 10 patients, including two in sinus rhythm, two with paroxysmal AF, three with persistent AF, and three with permanent AF. Immunohistochemical analysis of CD3-positive T cells in the tissue samples revealed that the number of inflammatory CD3-positive T cells varied among patients with different states of AF. Specifically, the mean number of CD3-positive T cells per 1 mm^2^ was 0.27 in the sinus rhythm group, 1.55 in patients with paroxysmal AF, 2.38 in the persistent AF subgroup, and 1.28 in patients with permanent AF.^[38]^ This seems to indicate that CD3-positive T cells play an important role in the development of AF. This is consistent with our experimental results. Our experiments found that the level of CD3 expression on terminally differentiated CD4^+^ T cells was positively correlated with AF (OR: 1.0285, 95% CI: 1.0065–1.0509; *P* = .0107). In an observational study of 100 consecutive patients, including 80 patients with a first diagnosis of AF and 20 control patients, it was found that pro-inflammatory and cytotoxic T-lymphocyte subpopulations (CD8+) circulate more frequently during early AF compared with patients without AF and are accompanied by increased levels of plasma CD8^+^ effector molecules.^[18]^ Activated CD8^+^ T cells perform by releasing cytotoxins (perforin, granzyme and granulysin) or by releasing the secretion of pro-inflammatory cytokines, such as TNF-α and IFN-γ to induce apoptosis in target cells.^[39,40]^

Peripheral monocytes trigger an inflammatory cascade that releases TGF-β, ROS, complement proteins, and chemokines, ultimately contributing to cardiac fibrosis. Among these factors, monocyte chemoattractant protein-1 and its receptor CCR2 play a pivotal role in this process.^[41,42]^ We determined that CD40 expressed on monocytes contributes to the progression of AF (OR: 1.0149, 95% CI: 1.0035–1.0264; *P* = .0101). Regulatory T cells inhibit the activation and proliferation of effector T cells, thereby controlling the immune response.^[43]^ Previous studies have found that the proportion of regulatory T cells is significantly lower in patients with AF.^[44]^ Our study found that Activated & resting CD4 regulatory T cell Absolute Count has a protective effect against AF (OR: 0.9513, 95% CI: 0.9165–0.9874; *P* = .0086). Nevertheless, the precise processes by which AF triggers inflammation are incompletely comprehended.

Several constraints exist in our study that necessitate consideration. First, although multiple sensitivity experiments have been conducted, a thorough assessment of horizontal pleiotropy is still difficult to exclude. Second, the lack of individual-level data impedes our ability to perform more detailed analysis that is segmented into specific subgroups within the population. Third, our study relied on a European database, so restricting the generalizability of our findings to other ethnic populations and diminishing the feasibility of our conclusions. Fourth, we did not employ multiple trial adjustments based on the False Discovery Rate method, potentially leading to some false positive results. More research is needed to find out the link between the immune system and AF and the specific mechanisms involved.

## 5. Conclusion

Utilizing bidirectional MR analysis, we investigated the causal association between the immune system and AF while elucidating their underlying mechanisms. Furthermore, our study mitigated the influence of confounders, reverse causality, and other variables, potentially facilitating research into the biological mechanisms of AF and the discovery of novel therapeutic strategies.

## Acknowledgments

The authors extend their gratitude to all participants of this MR study, as well as the Finngen database for providing the published GWAS summary statistics.

## Author contributions

**Data curation:** Xia Guo.

**Investigation:** Haoxuan Chu, Xia Guo, Hanchi Xu.

**Methodology:** Haoxuan Chu, Hanchi Xu.

**Resources:** Shipeng Wang.

**Software:** Haoxuan Chu, Xia Guo.

**Writing – original draft:** Haoxuan Chu.

**Writing – review & editing:** Jiahuan He, Yushi Wang.

## Supplementary Material





**Figure:**